# Why stay in a bad relationship? The effect of local host phenology on a generalist butterfly feeding on a low-ranked host

**DOI:** 10.1186/s12862-016-0709-x

**Published:** 2016-06-29

**Authors:** Hélène Audusseau, Maria de la Paz Celorio-Mancera, Niklas Janz, Sören Nylin

**Affiliations:** UMR Institute of Ecology and Environmental Sciences-Paris, Paris-Est Créteil University, Créteil, France; Department of Zoology, Stockholm University, Stockholm, Sweden

**Keywords:** Adaptation, GC-MS, Host plant range, Larval performance, LC-MS, Metabolomics, Oviposition preference, Plasticity, Primary and secondary metabolites, *Vanessa cardui*

## Abstract

**Background:**

In plant-feeding insects, the evolutionary retention of polyphagy remains puzzling. A better understanding of the relationship between these organisms and changes in the metabolome of their host plants is likely to suggest functional links between them, and may provide insights into how polyphagy is maintained.

**Results:**

We investigated the phenological change of *Cynoglossum officinale*, and how a generalist butterfly species, *Vanessa cardui*, responded to this change. We used untargeted metabolite profiling to map plant seasonal changes in both primary and secondary metabolites. We compared these data to differences in larval performance on vegetative plants early and late in the season. We also performed two oviposition preference experiments to test females’ ability to choose between plant developmental stages (vegetative and reproductive) early and late in the season. We found clear seasonal changes in plant primary and secondary metabolites that correlated with larval performance. The seasonal change in plant metabolome reflected changes in both nutrition and toxicity and resulted in zero survival in the late period. However, large differences among families in larval ability to feed on *C. officinale* suggest that there is genetic variation for performance on this host. Moreover, females accepted all plants for oviposition, and were not able to discriminate between plant developmental stages, in spite of the observed overall differences in metabolite profile potentially associated with differences in suitability as larval food.

**Conclusions:**

In *V. cardui*, migratory behavior, and thus larval feeding times, are not synchronized with plant phenology at the reproductive site*.* This lack of synchronization, coupled with the observed lack of discriminatory oviposition, obviously has potential fitness costs. However, this “opportunistic” behavior may as well function as a source of potential host plant evolution, promoting for example the acceptance of new plants.

**Electronic supplementary material:**

The online version of this article (doi:10.1186/s12862-016-0709-x) contains supplementary material, which is available to authorized users.

## Background

The diversification of plant chemicals has long been seen as an important explanation for the species richness of plant-feeding insects, as well as for their high degree of host specialization [12, 20]. For plant-feeding insects, this codiversification and their dependence on their resources have often necessitated the evolution of specific adaptations to the chemical composition of their hosts. Such adaptations should be even more demanding in generalist than in specialist species. Generalist larvae face a larger variability in chemical compounds across the host plant repertoire than specialist species [44], but their broad host range will also make it increasingly difficult for adult females to select optimal oviposition sites. For example, Janz and Nylin [21] showed that among a set of five nettle-feeding butterfly species, only specialists were able to reliably distinguish between leaves of different nutritional quality. As a consequence of this adult inability in generalist species to adequately determine individual leaf quality, a substantial number of offspring are likely to end up feeding on plant individuals that are poor hosts. Hence, the evolutionary retention of polyphagy remains puzzling.

Moreover, host plants are not only chemically different across species but plant species are also chemically highly heterogeneous both in space and time [13, 28, 33, 34, 37]. For example, seasonal changes in plant quality are seen at the molecular level through changes in both primary metabolites - corresponding to proteins, carbohydrates, and lipids [10] - and secondary metabolites, such as terpenoids, alkaloids and flavonoids. Seasonal changes are often the result of changes in resource allocation. Before flowering, plants are likely to allocate more resources to the production of reproductive organs rather than to the accumulation of secondary metabolites directed to energy-storage or herbivore defense [4, 23]. This high seasonal variability in plant quality can affect the fitness of the species that feed on them in several ways. Both fluctuations of primary metabolites, which are the main sources of nutrition for plant-feeding species, and of secondary metabolites, which are important components of plant defense against herbivory (e.g., [13, 15, 42]), interact with insect development and limit survival [41]. Thus, the temporal differences in resource allocation in plants may open windows of opportunity for insect species to exploit plants and complete their life cycle during a time-frame when plant chemical composition is most favorable for larval growth and development [3].

Hence, to feed on a wide number host plants, which are associated with unique seasonal changes in chemistry, generalist species have been shown to invest for instance in broad detoxification strategies, while specialist species restrict their adaptations to the metabolization of a narrower range of compounds [16, 24]. A better understanding of how generalists adapt to the chemical composition of their hosts could shed light on the evolution of host use in general. Most studies that have addressed this question have investigated the interaction between insects and selected plant compounds, known to interfere with larval development (*cf.* [26]). The development of new techniques allow us to go further. Untargeted study of changes in host plant chemicals, in combination with larval monitoring, may reveal a broader range of compounds involved in this ecological interaction, suggest functional links between the interacting species, and, ultimately, provide some insights into what makes species retain such a broad host plant range.

Here, we investigate the level of adaptation of a generalist butterfly species, *Vanessa cardui,* to one of its host plant, *Cynoglossum officinale* (Boraginaceae). The cosmopolitan migrant *V.cardui* (the painted lady) is possibly the most polyphagous of all butterflies [29], recorded from plants in ten angiosperm orders. Some of its hosts are shared with other close relatives [18], while the majority – such as plants in the family Boraginaceae – are recent colonizations by *V. cardui* [29]. *C. officinale* is a host low in its preference hierarchy [8, 11]. Interestingly, the suitability of this host to support larval growth drastically deteriorates as the season progresses. Anecdotal observations in Sweden, derived from previous rearing of *V. cardui* on *C. officinale* for an earlier study, have shown that larval offspring that feed on *C. officinale* early in the season (May-June) successfully reach adulthood [8]. Late in the season (July-August), development until adult emergence on this plant is rare [8]. Therefore, depending on arrival time, migrant females – and their larval offspring – will encounter *C. officinale* in various stages of phenological development, depending on the exact timing of the northward migration. Offspring of early migrants are likely to be able to use this host successfully during the entire development, while latecomers may suffer severe fitness costs, at least in late developmental stages.

Since plants are chemically complex and it can be difficult to determine *a priori* all chemical changes that will matter from the point of view of insect performance and survival, we used untargeted metabolite profiling to map seasonal changes in both primary and secondary metabolites. We investigated changes in *C. officinale* plant metabolomes according to their seasonal progression and developmental stage. We compared these data to larval performance on vegetative plants and hypothesized that the seasonal accumulation of secondary metabolites in *C. officinale* will induce a lower performance of the larvae. We also performed an oviposition preference experiment to test female ability to choose between plants in different developmental stages, at two different stages of phenological progression, in order to test if females are able to distinguish between plants with different suitabilities as larval food.

## Methods

### Insect material

The *V. cardui* population originated from larvae and pupae obtained from a commercial supplier in May 2014 (World Wide Butterflies, www.wwb.co.uk). This population was maintained under laboratory conditions (25 °C; 18 L:6D) and the received larvae were reared on *Cirsium arvense* (Asteraceae) to pupation. Once the adults emerged, they were placed in a cage for mating. The mated females and their offspring were used in the experiments described below.

### Oviposition preference test

Seventeen *V. cardui* mated females (cf. above) were used in an oviposition preference test between June 16^th^ and June 22^th^ (referred to as “early oviposition”). Females were kept in individual cages with the male they first mated with (for potential remating) at 27 ± 1.8 °C, under 9.5 L:14.5D light regime, with food (sugar solution) *ad libitum*, and humidity in the cage was maintained with wet paper on the floor. We performed a pairwise-choice test in which freshly collected leaves of approximately similar size, from vegetative or reproductive plants, were presented simultaneously to the females between 11:00 and 14:00. At the end of the trial, eggs laid on each leaf were counted and kept by family (or mother) at room temperature for consecutive larval rearing. The test was considered successful if the female laid a minimum of 10 eggs per day over two successful days (trials).

We performed this oviposition preference test again with ten adult offspring of these females between July 22^nd^ and July 29^th^ (referred to as “late oviposition”) using the exact same protocol as the one described above (early in the season). These two oviposition preference tests were performed to investigate female ability to discriminate between *C. officinale* developmental stages and if this ability changed with season.

### Larval rearing

Eggs collected from the early and late oviposition preference tests were consecutively used in two larval rearings (referred to as “early rearing” and “late rearing”, respectively). Upon hatching, larvae were reared in a split-brood design at room temperature on both artificial diet (as control, Stonefly Heliothis diet from Ward’s Science, Rochester, NY) and on freshly collected leaves from vegetative plants of *C. officinale* with a 1:3 initial ratio with respect to these diets (we aimed for five larvae feeding on the artificial diet and fifteen on *C. officinale* to account for expected differences in survival [8]). While plants were expected to change in quality as food between the two rearing events, the artificial diet allowed us to control for seasonal differences in chemical composition in the food, which we hypothesized to affect larval development. Therefore, larvae feeding on the artificial diet should perform similarly in the early and late rearing while we expected a seasonal decrease in performance of larvae feeding on *C. officinale.* These two rearings allowed us to assess larval performance of feeding on *C. officinale* in interaction with plant seasonality.

Over the two larval rearings, we monitored pupal mass, developmental time to adult emergence and survival rate after successful larval establishment (monitored after larval third instar). In the “early rearing” we reared 312 larvae from 17 different families and in the “late rearing” 121 larvae from 12 different families. During the late rearing, we also recorded food intake by weighing caterpillar frass of a subsample of individuals feeding on artificial diet and on *C. officinale.* Frass of three individuals of newly molted third instar larvae of the same family reared on artificial diet and on *C. officinale* was collected over a two-day period. Frass was dried for 20 h at 50 °C and weighed.

### Plant material and metabolite profiling

Plant material from *C. officinale* was collected, at the time of the oviposition preference tests and larval rearings, to investigate seasonal changes in plant primary and secondary metabolites that could affect female oviposition preference and larval performance*.* Most leaves were collected from the field around Stockholm University (Sweden) except in the early rearing, for which we also used plants growing in the laboratory because of limitation of field resources to support the rearing of a large population. Specifically, we collected 180 samples of leaf tissue (Table 1). Throughout each of the two oviposition preference tests we sampled three replicates (three leaves) from each of ten vegetative plants and three replicates from each of ten reproductive plants that were proposed to the females (Table 1). In the same way, throughout each of the two larval rearings we sampled three replicates from each of ten vegetative plants that were used as food for larvae (Table 1). In the early rearing, eight of the ten vegetative plants were sampled from the field and two from the laboratory in order to control for differences in plant chemical composition between growing conditions which may have affected larval performance. In the late rearing, all plants were sampled from the field.

**Table 1:** Number of leaf tissue of vegetative and reproductive plants sampled in the oviposition preference tests and larval rearings, early and late in the season

Sample size N (leaves per plants x no. of plants)		Vegetative plants		Reproductive plants
		Field	Laboratory	Field
Seasonal progression	Early oviposition (June 16^th^ to 22^nd^)	30 (3 × 10)	–	30 (3 × 10)
	Early rearing (June 20^th^ to July 21^st^)	24 (3 × 8)	6 (3 × 2)	–
	Late oviposition (July 22^th^ to 29^th^)	30 (3 × 10)	–	30 (3 × 10)
	Late rearing (July 28^th^ to August 16^th^)	30 (3 × 10)	–	–
Total		120		60

All reproductive plants were sampled from the field. A subsample of the vegetative plants sampled during the early rearing grew in the laboratory

Approximately 200 mg of each leaf tissue sampled was frozen in liquid nitrogen. The leaf samples were ground under liquid nitrogen with mortar and pestle and stored in −80 °C until analysis. In collaboration with the Swedish Metabolomics Center (SMC) in Umeå we obtained an untargeted metabolite profile of the plant samples. Gas Chromatography (GC) and Liquid Chromatography (LC) were coupled with Mass Spectrophotometry (MS) for metabolite profiling. These two techniques allow analyzing compounds with low and high polarity that broadly correspond to primary and secondary metabolites, respectively. The details on the GC-MS and LC-MS (both in positive and negative mode) methods, the quality control of internal standards, and identification of compounds are provided in the Additional file 1.

### Statistical analysis

First, we investigated differences in metabolite profile (i) between vegetative plants used in the two larval rearings, (ii) of each plant developmental stage used in the oviposition preference experiments over the season, and (iii) between plant developmental stages used in each of the oviposition preference tests. Plants used in the larval rearings were sampled just after collection from the field whereas plants used in the oviposition preference experiments were kept in water for the time of the oviposition trial before sampling. This difference in plant conditioning between plants used in the larval rearings and oviposition preference tests did not allow us to compare vegetative plants used in both set-ups.

Analyses were performed separately for data obtained from GC-MS, LC-MS positive mode, and LC-MS negative mode. Investigations of data structuration were carried out using two multivariate statistics, the principal component analysis (PCA) and the orthogonal partial least squares discriminant analysis (OPLS-DA), available in the software package SIMCA version 13.0.2 (Umetrics, Umeå, Sweden). The PCA was used for data overview, and to detect trends and outliers. OPLS-DA was used to find the compounds that were most related to the specified class discrimination. The fit of the models was estimated based on the R^2^ values (R^2^X and R^2^Y) and the predictive capability parameter (Q^2^) determined through a 7-fold cross validation. The significance of the OPLS-DA models were estimated based on the results from an ANOVA of the cross-validated predictive residuals (CV-ANOVA), the CV-scores, and permutation tests (*n* = 999). For each model using OPLS-DA, compounds were considered discriminant based on their statistical difference at the same time in OPLS-DA, univariate t-tests at a 95 % confidence level with Benjamini-Hochberg correction, and Mann-Withney U test. All calculations were performed in MATLAB® (MathWorks®, Natick, USA). GC-MS data were used in the raw format, as normalization did not improve the analyses. In both the positive and negative modes, LC-MS data were normalized by the scores of a PCA on the matrix the internal standards. All models used UV-scale data to weight the variables.

Second, we analyzed differences in larval performance in terms of larval growth, ability to feed, and survival rate. For individuals that successfully reached adult emergence, we tested for the effect of diet and seasonality on larval growth rate (*cf.* eq.1) using an ANOVA. The two-way interaction between diet and seasonality could not be tested since none of the larvae feeding on *C. officinale* in the late rearing survived to emergence. Family could also not be included, as for many families only one individual reached emergence. An ANOVA was also used to investigate the effect of diet, family, and their interaction, and individual subsequent survival on frass weight. The analysis was performed on the logarithm of frass weight to fit the assumptions of the linear model. Next, we tested for the effect of diet, seasonality and the two-way interaction on larval survival rates. The response variable used for the analysis of survival was a two-level factor of the number of individuals that survived and the number of individuals that died per family. We performed a generalized linear model using a quasibinomial distribution to account for overdispersion. When included in the models, family was considered as a fixed effect to identify potential differences between families.

1 $$ \mathrm{Growth}\ \mathrm{Rate} = \ln \left(\mathrm{pupal}\ \mathrm{weight}\right)/\mathrm{Time}\ \mathrm{t}\mathrm{o}\ \mathrm{emergence} $$

Last, we investigated the ability of ovipositing *V. cardui* females to discriminate between vegetative and reproductive stages of *C. officinale*. We analyzed female oviposition preference according to plant developmental stage and seasonality performing a Friedman-rank test in two different manners. First, using the ranking of the total number of eggs laid by a female on either vegetative or reproductive leaves of *C. officinale* across the two-day trial. Second, using the ranking of the summed scores assigned to each female per daily oviposition trial. That is, for each day, the value one was given to the preferred plant and the value zero for the less preferred one (with a difference in eggs laid of a minimum of two eggs). The summed scores corresponded to the sum of scores given to each plant developmental stage over the two-day trial for each female (giving a maximum value of two). All analyses of larval performance and female oviposition preference were performed in R version R.3.1.1 [31].

## Results

### Plant metabolite profile

Across the 180 plant samples, the GC-MS techniques allowed for the quantification of 61 compounds, among which 41 were identified, names were suggested for 7, and the 7 remaining were unidentified. Using the LC-MS method, we quantified 3979 compounds, 1780 in the positive mode and 2199 in the negative mode. Only 61 are so far identified.

**Fig. 1:**
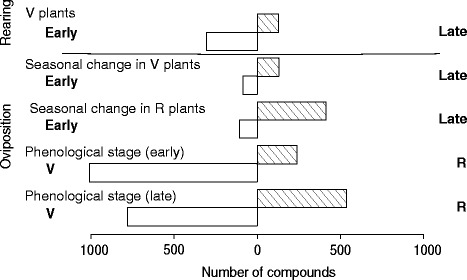
Number of compounds that were significantly more present in one or the other plant group from the analyses of class discrimination (i) between vegetative plants used in the two larval rearing (early on the right vs late on the left), (ii) of each plant developmental stage used in the oviposition preference tests over the season (seasonal change in vegetative (V) and reproductive (R) plants), and (iii) between plant developmental stages used in each of the oviposition preference tests (developmental stage V versus R early and late)

All OPLS-DA models were validated (Table 2). Obvious class separation was detected in those metabolite profiles between early and late vegetative plants used in the larval rearing (Fig. 1, Table 2). 433 metabolites were significantly discriminant between vegetative plants sampled early and late in the season. Among the compounds identified, we found a significant seasonal downward trend in organic acids, fatty acid and lipids, and some amino acids such as for alendronate, valine and tyrosine (Table 3). On the other hand, some compounds significantly accumulated, as was the case for GABA (4-aminobutyric acid). Moreover, vegetative plants grown in the laboratory did not differ from vegetative plants collected in the field.

**Table 2:** Statistics of the OPLS-DA models used to investigate class discrimination (i) between vegetative plants used in the two larval rearing (V plants early vs late), (ii) of each plant developmental stage used in the oviposition preference tests over the season (V plants early vs late, R plants early vs late), and (iii) between plant developmental stage used in each of the oviposition preference tests (V vs R in Ovip early and late)

Models			GC-MS						LC-MS positive mode						LC-MS negative mode					
			Models statistics				Model validation		Models statistics				Model validation		Models statistics				Model validation	
			Nob.	R^2^X	R^2^Y	Q^2^	CV-ANOVA	Intercept	Nob.	R^2^X	R^2^Y	Q^2^	CV-ANOVA	Intercept	Nob.	R^2^X	R^2^Y	Q^2^	CV-ANOVA	Intercept
Class discrimination between *C. officinale* plants used in:	Rearing	V plants early vs late	1 + 1	0.28	0.76	0.60	2.4 e^−10^	−0.379	1 + 1	0.21	0.85	0.72	1.1e^−13^	−0.45	1 + 1	0.19	0.76	0.61	1.3 e^−10^	−0.39
	Oviposition preference	V plants Early vs late	1 + 1	0.28	0.64	0.44	1.6 e^−6^	−0.397	1 + 1	0.16	0.84	0.65	4.8 e^−12^	−0.416	1 + 2	0.39	0.79	0.61	2.3 e^−9^	−0.455
		R plants Early vs late	1 + 1	0.43	0.92	0.90	2.4 e^−25^	−0.376	1 + 1	0.24	0.93	0.88	5.5 e^−24^	−0.445	1 + 1	0.36	0.91	0.86	1.0 e^−22^	−0.406
		Ovip early V vs R	1	0.19	0.64	0.58	3.2 e^−11^	−0.258	1 + 1	0.22	0.80	0.58	2.5 e^−9^	−0.387	1	0.17	0.64	0.55	1.1 e^−10^	−0.295
		Ovip late V vs R	1 + 1	0.38	0.91	0.86	1.7 e^−22^	−0.382	1 + 1	0.26	0.91	0.82	3.4 e^−19^	−0.433	1 + 1	0.26	0.88	0.79	1.3 e^−17^	−0.402

For each model are shown the number of component of the model, the variance captured by the model (R^2^X), the y-fit (R^2^Y), and the predictive capability (Q^2^). The significance of the OPLS-DA models were evaluated based on the *p*-value of the Cross-validation ANOVA, and the intercept obtained from the permutation test. R and V correspond to reproductive and vegetative plants, respectively

**Table 3:** List of metabolites that significantly vary in one of the analyses performed to investigate class discrimination (i) between vegetative plants used in the two larval rearing (V plants early vs late), (ii) of each plant developmental stage used in the oviposition preference tests over the season (V plants early vs late, R plants early vs late), and (iii) between plant developmental stages used in each of the oviposition preference tests (V vs R in Ovip early and late)

	Rearing		Oviposition		
Names	V plants Early vs late	V plants Early vs late	R plants Early vs late	Ovip early V vs R	Ovip late V vs R
**Primary metabolites**					
**Sugars and sugar alcohols**					
Beta-d-lactose	ns	ns	ns	ns	+
Cellobiose	ns	ns	+	ns	+
D-Glucoheptose	−	ns	+	ns	+
Fructose (1 & 2)	ns	ns	+	ns	+
Fructose-6-phosphate	+	ns	−	ns	−
Glucose (1 & 2)	ns	ns	ns	ns	+
Glucose-6-phosphate	+	ns	−	ns	−
Glycerol-3-phosphate	ns	ns	ns	−	−
Inositol, scyllo	ns	ns	ns	ns	−
Inositol-1-phosphate, myo-like	+	ns	ns	−	−
Lactitol	−	ns	ns	+	+
Lactitol 1	ns	ns	+	+	+
Laminaribiose	ns	ns	+	ns	+
L-Arabinose	ns	ns	+	ns	+
Maltitol	−	ns	+	ns	+
Maltotriose 1	−	−	ns	ns	ns
Maltotriose 2	ns	ns	−	+	ns
Maltotriose 3	ns	ns	+	ns	+
Mannitol	ns	+	+	ns	ns
Ononitol-like	ns	ns	ns	−	−
Ribose	+	ns	+	−	ns
Sucrose-8TMS M/z450	ns	−	ns	−	ns
Trehalose M/z191	ns	ns	+	ns	+
Xylitol	−	ns	+	ns	+
**Organic acids**					
Alpha-ketoglutaric acid	ns	ns	+	−	ns
Citric acid	−	ns	+	−	+
Citric acid 1	ns	ns	+	−	ns
Citric acid 2	ns	ns	+	−	+
Dehydroascorbic acid dimer	ns	−	−	−	−
Fumaric acid	ns	ns	+	−	ns
Glucoheptonic acid	ns	ns	ns	ns	+
Gluconic acid	−	ns	+	ns	+
Glyceric acid	−	−	ns	−	ns
L-2-Aminoadipic acid	ns	ns	ns	–	ns
L-Ascorbic acid	ns	ns	ns	–	ns
Malic acid	–	ns	+	–	ns
Malic acid (C_4_-DC-OH) 1	–	ns	+	–	+
Niacin (Nicotinic acid)	ns	ns	ns	–	–
Threonic acid-like	–	–	+	–	+
**Fatty acids and lipids**					
Erythronic acid-like	–	ns	+	ns	+
Galactonic acid-like	–	ns	+	ns	+
Linoleic acid (18:2)	ns	ns	+	ns	ns
Palmitic acid (16:0)	ns	ns	+	ns	ns
Pentonic acid-like	–	ns	+	–	+
Threonate	–	ns	+	–	+
**Inorganic and mineral compounds**					
Phosphate-fragment	+	ns	–	ns	–
Phosphoric acid	+	ns	ns	ns	ns
**Protein amino acids and cofactors**					
Alanine	ns	ns	+	ns	ns
Alendronate	–	ns	ns	ns	+
Asparagine	ns	ns	ns	–	–
Aspartic acid	ns	ns	ns	–	–
D-Glutamate	ns	ns	ns	–	–
D-Glutamate 1	ns	ns	ns	ns	+
D-Glutamate 2	ns	ns	ns	–	ns
DL-Serine	+	+	ns	ns	ns
DL-Valine 3	ns	ns	ns	ns	–
DL-Valine 4	–	ns	ns	ns	+
Eicosanoic acid	ns	ns	+	+	+
Galactosylglycerol-like	ns	ns	–	+	+
Gamma-tocopherol	+	ns	–		–
Glutamic acid	ns	ns	+	–	–
Glutamine	ns	ns	+	ns	+
Leucine	ns	ns	–	ns	ns
L-Tryptophan [M-NH_3_]	ns	ns	ns	ns	+
Phenylalanine (1 & 2)	ns	ns	ns	ns	+
Succinic acid	ns	ns	+	–	+
Succinic acid (C_4_-DC)	ns	ns	+	ns	+
Tryptophan M/z291	ns	ns	ns	ns	+
Tyrosine	–	ns	ns	ns	ns
Uridine	+	+	+	–	–
**Other nitrogen-containing metabolites**					
4-aminobutyric acid (GABA)	+	ns	ns	ns	ns
Allantoin	+	ns	ns	–	–
Hypoxanthine	ns	ns	ns	–	+
Adenine	ns	ns	+	–	ns
a-N-Acetylglucosamine	ns	ns	ns	–	ns
**Secondary metabolites**					
C_10_H_12_O_7_S	–	ns	+	ns	+
C_15_H_28_O_15_	–	–	ns	ns	+
C_17_H_18_N_4_O_5_	–	ns	+	ns	+
C_19_H_33_NO_11_	–	ns	+	+	+
C_19_H_34_N_2_O_8_S	–	ns	+	ns	+
C_20_H_34_O_10_	–	ns	+	ns	ns
C_20_H_34_O_11_	–	ns	+	+	+
C_20_H_8_N_2_O_4_	–	ns	+	ns	ns
C_22_H_30_N_6_O_10_S_2_	–	ns	+	+	+
C_27_H_36_O_14_	–	ns	+	+	+
C_28_H_33_N_5_O_5_S_2_	–	ns	+	+	+
C_33_H_30_O_17_	–	–	ns	ns	ns
C_38_H_40_N_2_O_10_	–	ns	+	–	+
C_38_H_50_O_11_S_2_	–	ns	+	+	+
Carbohydrate (C_20_H_30_O_15_)	–	ns	+	+	+
Carbohydrate acid (C_9_H_16_O_9_)	–	ns	+	ns	+
Carboxylic acid (C_3_H_6_O_4_), Glyceric acid	+	+	ns	ns	ns
Carboxylic acid (C_6_H_10_O5)	–	ns	+	ns	ns
Carboxylic acid (C_6_H_12_O3)	–	ns	+	–	ns
Carboxylic acid (C_6_H_12_O7)	–	ns	+	ns	+
Flavonoid	–	ns	–	–	ns
Flavonoid (C_15_H_16_O_9_)	–	ns	+	ns	ns
Flavonoid (C_27_H_30_O_16_), Rutin	–	ns	–	ns	ns
Lignan	–	ns	ns	+	ns
Lignan (C_22_H_26_O_8_)	–	ns	ns	+	ns
Phenolic glycoside (C_12_H_18_O_11_)	–	ns	+	ns	+
Phenolic glycoside (C_14_H_18_O_9_)	–	ns	ns	ns	ns
Phenolic glycoside (C_14_H_18_O_9_)	–	ns	ns	ns	ns
Phenolic glycoside (C_14_H_18_O_9_)	–	ns	+	ns	+
Phenolic glycoside (C_17_H_26_O_12_)	–	ns	+	+	+
Phenolic glycoside (C_17_H_26_O_12_)	–	ns	+	+	+
Phenolic glycoside (C_17_H_26_O_12_)	–	ns	+	+	+
Phenolic glycoside (C_18_H_30_O_10_)	–	ns	+	ns	+
Phenolic glycoside (C_26_H_28_O_8_)	–	ns	+	+	+
Phenolic glycoside (C_26_H_30_O_9_)	–	ns	+	+	+
Phenolic glycoside (C_26_H_32_O_11_)	–	ns	+	+	+
Phenolic glycoside (C_27_H_34_O_13_)	–	ns	+	+	+
Phenolic glycoside (C_28_H_26_O_6_)	–	ns	+	+	+
Phenolic glycoside (C_28_H_26_O_7_)	–	ns	+	+	+
Phenolic glycoside (C_32_H_30_O_10_)	–	ns	+	+	+
Phenolic glycoside (C_32_H_42_O_16_)	–	ns	+	–	+
Rutin 1	–	ns	–	ns	ns
Rutin 2	ns	–	ns	–	ns

Compounds were considered discriminant based on their statistical difference in OPLS-DA, univariate t-tests (Benjamini-Hochberg correction), and Mann-Withney U test (*Cf.* Methods for details). “+” indicates compounds that were more abundant in the second called group, “-” indicates compounds that were more abundant in the first group, “ns” are compounds that did not significantly vary between classes. For example, ribose was more abundant in vegetative plants from the late rearing. The data in boldface are the categories of compounds

Class separations were also clear between plant developmental stages when analyzing samples of the plants used in each of the two oviposition preference tests (Fig. 1, Table 2). The highest difference between classes, in terms of number of compounds that significantly varied, was found between vegetative and reproductive plants, both early and late in the season. Among the metabolites they shared, a greater number was more abundant in vegetative plants than in reproductive plants (Fig. 1, Table 3). We also found differences within each plant developmental stage over the season. Seasonal change was more pronounced for reproductive plants than for vegetative plants, with a significant seasonal change in the relative abundance of 521 and 216 compounds, respectively (Fig. 1).

### Larval performance

Larvae feeding on the artificial diet grew significantly faster compared to larvae feeding on *C. officinale* (diet: F_(1,117)_ = 77.2, *p* < 0.001, see Additional file 2, Fig. 2a)*.* This difference was mainly a consequence of differences in final mass (by a factor 3; in the early rearing mean ± sd of pupal mass = 625.9 ± 63.2 mg on artificial diet and 241.9 ± 32.6 on *C. officinale*) as development time to emergence on both treatments were similar (in the early rearing mean ± sd of pupal time = 16.3 ± 2.4 days on artificial diet and 17.6 ± 1.0 on *C. officinale*).

**Fig. 2:**
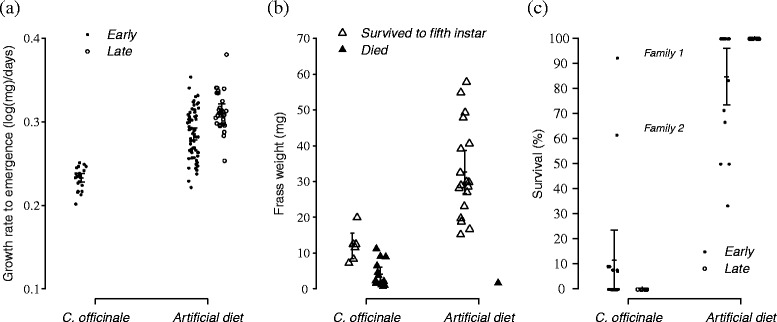
**a** Growth rate to emergence (average ln(mg).day^−1^ ± CI) according to food diet (artificial diet and *C. officinale*) and seasonality. The points represent individuals’ growth. **b** Frass weight (average mg ± CI) of third instar larvae that have been feeding for two consecutive days on artificial diet or *C. officinale* and according to their survival a posteriori. The triangles correspond to the value for each individual. **c** Survival of larvae feeding on artificial diet and *C. officinale* early and late in the season (average survival in % ± CI). The circles correspond to the average survival in each family

Frass weight varied significantly according to the diet in the late rearing (diet: F_(1,22)_ = 36.6, *p* < 0.001, see Additional file 3) and was 2.7 higher for larvae feeding on artificial diet than on *C. officinale* (Fig. 2b).

In addition, survival rates were higher for larvae feeding on artificial diet compared to larvae feeding on plants (diet: LR Chisq _(1, N=47)_ = 82.91, *p* < 0.001, see Additional file 4). Consistent with our previous observations, larvae reared on *C. officinale* early in the season had better survival than larvae reared late in the season, during which none of the larvae survived to adult emergence (Fig. 2c). We also found a significant interaction between seasonality and diet which was somewhat unexpected as it resulted from a seasonal difference in survival of larvae feeding on artificial diet (diet x seasonality: LR Chisq _(1, N=47)_ = 8.43, *p* < 0.01, see Additional file 4, Fig. 2c). Most interestingly, the overdispersion in the model of survival was due to the comparably high survival of larvae of two families feeding on *C. officinale* (Fig. 2c). Early in the season, family 1 and 2 showed high survival rates of 92 and 61 %, respectively, in comparison with the others 15 families (Fig. 2c). Differences between families were also observed late in the season in the analysis of caterpillar frass (Fig. 2b). We found a significant effect of the interaction between family and diet on frass weight (diet x family: F_(1,22)_ = 3.2, *p* = 0.020, see Additional file 3). Specifically, one individual in family 23 feeding on *C. offinale* had a high frass weight comparable to frass weight from larvae feeding on artificial diet.

### Oviposition preference

Females showed no oviposition preference for vegetative or reproductive plants in any of the experiments and accepted all plants for oviposition, regardless of developmental stage (Table 4). They laid on average 62 ± 43 eggs per day, ranging from 10 to 169 eggs and from 11 to 260 eggs in the early and late oviposition preference tests, respectively. The two different methods used for the ranking in the Friedman test did not affect the results (Table 4).

**Table 4:** Friedman-rank statistics testing whether *V. cardui* mated females can discriminate between the developmental stages of *C. officinale*, early and late in the season, based on their oviposition preference on the plant leaves

	N	Chi Sq	df	*P*
Total egg count per female:				
Early V vs R	17	1.67	1	0.20
Late V vs R	10	0	1	1
Total scores per female:				
Early V vs R	17	2.27	1	0.13
Late V vs R	10	1	1	0.32

The table provides the results of the tests performed using ranks based on total egg-count per female and total preference scores per female

## Discussion

Data from plant metabolite profiles showed clear class separation according to plant developmental stage and seasonality (Fig. 1). Looking at vegetative plants (the stage used in the larval rearing), we examined plant seasonal changes in nutritional status and toxicity that could potentially explain the lower performance of the second generation of *V. cardui* offspring. The categorization of the metabolites seen in Table 3 should not be understood as definitive but rather as an attempt to identify broad patterns. This notwithstanding, the downward trend in amino acids likely translates into a decrease in nitrogen availability in the plants [25], which is known to be a limiting resource in insects [7, 27]. Seasonal variation in individual compounds may simply be added to this general decline to affect larval development. In addition, plant toxicity has likely increased through the season as seen in the significant increase in GABA. This plant compound has been proposed to act as an inhibitor of insect neuromuscular activity, leading to a reduction of larval growth and survival [6, 32].

Data from non-targeted metabolomics analyses should be considered carefully. The enormous amount of data generated from such analyses increases the probability to find random correlations between certain chemical compounds and insect performance, which necessitates further genetic studies to explore seasonal variation in a selected set of compounds and the corresponding gene expression in larvae. For example, based on personal observations made along larval rearings, we believe that some compounds accumulating through the season may interfere with pupation. Additionally, beside plants chemistry, physical parameters such as leaf thickness and toughness are known to interfere with larval feeding and female oviposition choice (e.g., [2, 9]). This being said, the lower fitness of the second generation of *V. cardui* offspring still correlated both with the observed decrease in plant nutritional status and increased toxicity. Indeed, while some of the larvae reared on *C. officinale* early in the season survived, late in the season none of the larvae reached adult emergence. While we could not correlate growth rate to food intake - individual frass was only collected during the second rearing where no measure of growth rate could be calculated as larvae died on the plant diet - we can still say that the differences in growth rate are likely caused by differences between diets in food intake. Interestingly, we also observed large differences across families in larval survival rate and feeding ability on *C. officinale*. Early in the season, family 1 and 2 reached 92 and 61 % survival, respectively. Late in the season, whereas the survival was null, one individual was feeding on *C. officinale*. Based on the measure of caterpillar frass, this individual ate a comparable amount to larvae feeding on artificial diet.

Moreover, we generally found that larvae feeding on *C. officinale* had lower growth rates and reduced survival rates compared to larvae feeding on the artificial diet. This was expected since *C. officinale* is a poor quality host and the artificial diet is a standardized mix of nutrients for optimal growth of Lepidoptera species. The difference in survival on artificial diet early and late in the season was more surprising since the chemical composition of the food was identical between the two rearing events. However, rearing was done at room temperature, which is likely to have increased during the particularly warm summer in 2014 (outside temperature: T_mean June_ = 13.7 °C, T_mean July_ = 19.4 °C). Still, if rearing conditions were more favorable late in the season for larvae feeding on the artificial diet, it makes the extremely poor larval survival on late-season *C. officinale* even more striking.

In both oviposition preference tests, females laid significant amounts of eggs on *C. officinale* and showed no preference between vegetative and reproductive plants, in spite of their observed overall metabolome differences and differences in suitability as larval food (Fig. 1). The higher seasonal change in reproductive plants compared to vegetative plants (in terms of number of compounds that significantly varied in abundance over the seasons) could result in a lower suitability of reproductive plants as larval food. However, support for differences in suitability between plant developmental stages is still pending further identification of the chemical compounds quantified and work on their role in insect-plant interactions. In any case, the lack of discriminatory behavior in adult females may have serious fitness costs for the offspring. This is concordant with previous findings that also showed a lack of discrimination between intraspecific plants that differed in quality [21, 36]. Although preference and performance across hosts is not well-studied in this species, it does appear that *V. cardui* discriminates between host species and has a preference hierarchy [19, 38]. It has been suggested that the ability of generalists to discriminate between large numbers of host species may negatively affect the more fine-grained discrimination required for detecting intraspecific differences in quality [5, 21]. Still, when the failure to discriminate against a poor host is as severe as it appears to be in *V. cardui* on *C. officinale*, one may ask why the plant has not simply been dropped from the repertoire.

Part of the answer probably lies in the migratory behavior of *V. cardui*. The synchrony between female oviposition time and plant phenology is a key parameter for successful growth and survival on *C. officinale*, since larvae need to feed early in the season. Yet, as northward migration in *V. cardui* appears to be triggered by conditions in the source areas and prevailing winds [30, 39], the timing of arrival in Sweden is largely decoupled from the local seasonal progression. It is indeed likely that the preference hierarchy of *V. cardui* is shaped as an average of the temporal and spatial variation in plant availability and quality that the species encounters across its vast geographical distribution, and during its migrations. Hence, being a migratory species, it would make sense to be able to take advantage of any host that happens to be locally abundant. There are also indications that the oviposition behavior of *V. cardui* is highly plastic and opportunistic, where actual host use is to a large extent dependent on local host availability, but also on proximity to adult feeding sites [19, 38, 40]. It is possible that there are conditions where *C. officinale* (and other similar plants in Boraginaceae) are important local hosts. If so, late-season *C. officinale* in Sweden can be seen as a population sink, but selection favoring avoidance may simply be too weak, so that it is swamped by selection in other spatial and temporal contexts. Such decoupling of preference and performance should also promote the acceptance of new plants, thus setting the stage for the evolution of new host associations [1, 14, 22]. Because of the large potential variation in host plant adaptation in *V. cardui* – across hosts, as well as across time and space – further studies of the genetic and plastic component of intra- and interpopulation variation in preference and performance are particularly promising to study the evolution of host use in action.

## Conclusions

The observed lower fitness of the second generation of *V. cardui* offspring feeding on *C. officinale,* in comparison with larvae feeding early in the season, may correlate both with the observed decrease in plant nutritional status and increased toxicity. Interestingly, the variation across families in larval ability to feed on *C. officinale* suggests the presence of genetic variation and thus room for further adaptation (cf. [43]), should conditions favor the use of this plant. In both oviposition preference tests, females laid significant amounts of eggs on *C. officinale* and showed no preference between vegetative and reproductive plants, in spite of their observed overall metabolome differences and differences in suitability as larval food. Consequently, host use in *V. cardui* can probably best be described as opportunistic [40]. Oviposition preference in this species seems to be a labile behavior not tightly associated with individual larval performance and survival. Still, in a longer perspective, such apparent decoupling of preference and performance can function as an important source of potential host plant evolution, especially since we also found indications of genetic variation in performance on this host.

Metabolomics has been used to study host plant-herbivore interactions in order to further our understanding of the functional link between interacting species [17, 26]. However, metabolomics can also be used to understand evolutionary patterns [35]. In the case of the evolution of host plant range, combining studies on the plant metabolome with gene expression in the herbivore may provide insights into the genomic basis of polyphagy.

## Additional files

Additional file 1: Metabolic profiling by GC-MS and LC-MS. (PDF 93 kb) Additional file 2: ANOVA table showing the effect of diet and seasonality on growth rate to emergence. (PDF 85 kb) Additional file 3: ANOVA table showing the effect of diet, family, and survival on frass weight. (PDF 93 kb) Additional file 4: ANOVA table showing the effect of diet, seasonality and the interaction between diet and seasonality on survival. (PDF 76 kb)
