# Supplementary material for: Why stay in a bad relationship? The effect of local host phenology on a generalist butterfly feeding on a low-ranked host
Source: BMC Evol Biol. 2016 Jun 29;16:144. doi: 10.1186/s12862-016-0709-x (PMC4928354; doi:10.1186/s12862-016-0709-x)
Supplement: Additional file 1: — Metabolic profiling by GC-MS and LC-MS. (PDF 93 kb) [file 12862_2016_709_MOESM1_ESM.pdf]

Additional files

Why stay in a bad relationship? The effect of local host phenology on a generalist butterfly feeding on a low-ranked host.

Audusseau H., de la Paz Celorio-Mancera M., Janz N., Nylin S.

Additional file 1. Metabolic profiling by GC-MS and LC-MS.

Sample preparation

For each of the 180 samples, 1000 µL of extraction buffer (chloroform/water/methanol, 20/20/60, v/v/v) including 16 internal standards (11 targeted the GC-MS and 5 the LC-MS) were added to 9 - 12 mg of plant material. Internal standards were injected in each the sample for quality controls and normalization. The sample was then homogenized and centrifuged at +4°C, 14 000 rpm, for 10 minutes. 200 µL of supernatant was transferred to a micro vial and solvents were evaporated. In the LC-MS analysis, the sample was re-suspended in 10 µL methanol and 10 µL water before the analysis.

GC-MS analysis

GC-MS technique requires analytes to be vaporized. Therefore, a derivatization was performed to analyze metabolites present in the sample that are insufficiently volatile in their native state. To that end, 30 µL of methoxyamine (15 µg/µL in pyridine) were added to the dry sample. The sample was homogenized for 10 minutes and then left to react in room temperature for 16 hours. Then, 30 µL of N-methyl-N-trimethylsilyl-trifluoroacetamide

(MSTFA) were added to the sample and after homogenization the sample was left to react for 1 hour at room temperature. Finally, 30 μL of methyl stearate (15 ng/μL in heptane) was added before analysis.

Peak finding and quantification was performed by injecting 1 μL of the derivatized sample in a split (1:5) mode into a GC-MS system consisting of, an Agilent 6890 gas chromatograph, and a Pegasus III time-of-flight mass spectrometer, GC/TOFMS (Leco Corp., St Joseph, MI, USA). Chromatography was performed using a 10 m x 0.18 mm fused silica capillary column with a chemically bonded 0.18 μm DB 5-MS UI stationary phase (J&W Scientific). Injection temperature was 270°C, the purge flow rate was 20 $mL.min^{-1}$ and the purge was turned on after 60 seconds. The gas flow rate through the column was 1 $mL.min^{-1}$, the column temperature was held at 70°C for 2 minutes, then increased by $40°C.min^{-1}$ to 320°C, and held there for 2 minutes. The column effluent was introduced into the ion source of the spectrophotometer. The transfer line and the ion source temperatures were 250°C and 200°C, respectively. Ions were generated by a 70 eV electron beam at an ionization current of 2.0 mA, and 30 spectra s^{-1} were recorded in the mass range m/z 50 - 800. The acceleration voltage was turned on after a solvent delay of 150 seconds. The detector voltage was 1500-2000 V.

Processing was performed using SMC_RDA v.3994, a Matlab script developed at the Swedish Metabolomics Centre in Umeå(SMC, Mathworks, Natick, MA, USA). Identification of compounds is based on comparison with mass spectra libraries as well as retention indices. The target processing algorithm utilizes in-house library mass-spectral profiles (in-house databases developed at the SMC) and retention indices to locate, evaluate and integrate compounds of interest. The un-target algorithm utilizes peak picking and curve resolution for integrations and identifications of profiles (using both commercial NIST and in-house libraries). The un-target algorithm works for both supervised (class separation) and unsupervised (covariance) cases. In the analysis n-alkane series (C8-C40) was used to determine

retention indices and methylstearate was used as a quality control.

LC-MS analysis

One after the other, the 180 samples were analyzed in positive and negative mode. The chromatographic separation was performed on an Agilent 1290 Infinity UHPLC-system (Agilent Technologies, Waldbronn, Germany). 2 µL of re-suspended aliquots were injected into an Acquity UPLC HSS T3, 2.1 x 50 mm, 1.8 µm C18 column in combination with a 2.1 mm x 5 mm, 1.8 µm VanGuard precolumn (Waters Corporation, Milford, MA, USA) held at 40°C. The gradient elution buffers were A (H_2O, 0.1 % formic acid) and B (75/25, acetonitrile/2-propanol, 0.1% formic acid) at a flow-rate of 0.5 $mL.min^{-1}$. The compounds were eluted with a linear gradient consisting of 0.1 - 10% B over 2 minutes. B was increased to 99% over 5 minutes and held at 99% for 2 minutes. Afterwards, B was decreased to 0.1% for 0.3 minutes and the flow-rate was increased to 0.8 $mL.min^{-1}$ for 0.5 minutes. These latter conditions were held for 0.9 minutes, after which the flow-rate was reduced to 0.5 $mL.min^{-1}$ for 0.1 minutes before the next injection.

The compounds were detected with an Agilent 6550 Q-TOF mass spectrometer equipped with a jet stream electrospray ion source operating in positive or negative ion mode. The settings were kept identical between the modes, with exception of the capillary voltage. A reference interface was connected for accurate mass measurements. The reference ions purine (4 µM) and HP-0921 (Hexakis(1H, 1H, 3H-tetrafluoropropoxy)phosphazine) (1 µM) were infused directly into the mass spectrophotometer at a flow rate of 0.05 $mL.min^{-1}$ for internal calibration, and the monitored ions were purine m/z 121.05 and m/z 119.03632 for the positive mode and HP-0921 m/z 922.0098 and m/z 966.000725 for the negative mode. The gas temperature was set to 150°C, the drying gas flow to 16 $L.min^{-1}$ and the nebulizer pressure 35 psig. The sheath gas temperature was set to 350°C and the sheath gas flow 11 $L.min^{-1}$. The capillary voltage was set to 4000 V in positive ion mode, and to 4000 V in

negative ion mode. The nozzle voltage was 300 V. The fragmentor voltage was 380 V, the skimmer 45 V and the OCT 1 RF Vpp 750 V. The collision energy was set to 0 V. The m/z range was 70 - 1700, and data was collected in centroid mode with an acquisition rate of 4 scans s^{-1} (1977 transients/spectrum). The diode array detector was set to scan the interval 190 - 640 nm with a step length of 2 nm and a slit width of 4 nm.

The peak areas of the internal standards were calculated using the Agilent Masshunter Profinder version B.06.00 (Agilent Technologies Inc., Santa Clara, CA, USA). The processing was performed on the QC-samples using batch recursive feature extraction algorithm with the Agilent software Masshunter Profinder version B.06.00. The extracted features were matched using Mass Profiler Professional 13.0 (Agilent Technologies Inc., Santa Clara, CA, USA) and preliminary identified using the Agilent software Masshunter ID-Browser version B.07.00 (Agilent Technologies Inc., Santa Clara, CA, USA) together with the in-house library developed at the SMC. The recursion file was imported back into Masshunter Profinder and used for Batch Targeted Feature Extraction on all samples.

Quality control of internal standard

In the GC-MS analysis, all internal standards were detected and their Relative Standard Deviation (RSD) in retention time was acceptable (RSD% between 8-20%), except for $[^{13}C_4]$ hexadecanoic acid and $[^{13}C_5]$ proline which showed a larger variation (>40%). In the LC-MS, the internal standards were detected as well and their mass accuracy was good. We recorded a very small drift in retention time of the IS for the first batch. In the second batch the retention time drift disappeared.

Origin of the chemicals

The reagents were purchased at Sigma Aldrich (St. Louis, MO, USA) and Restek (Bellefonte, PA, USA). The solvent were obtained from Fischer Scientific (Waltham, MA, USA),

Merck (Darmstadt, Germany), Sigma Aldrich (St. Louis, MO, USA), Thermo Scientific (Bellefonte, PA, USA), and VWR (Radnor, PA, USA).

Internal reference standard used in the GC-MS were acquired from Sigma (St. Louis, MO, USA). The reference and tuning standards in the LC-MS were purchased at Agilent Technologies (Santa Clara, CA, USA).

The internal standard using in the GC-MS consisted of 2-hydroxy-[$^{2}H_{6}$] benzoic acid, [$^{2}H_{4}$]-1,4-butane-diamine 2HCl, [$^{13}C_{6}$] glucose, [$^{13}C_{4}$] hexadecanoic acid, [$^{13}C_{12}$] sucrose, [$^{2}H_{4}$] succinic acid and [$^{13}C_{5}$, ^{15}N] glutamic acid purchased from Sigma (St. Louis, MO, USA) and [$^{2}H_{7}$] cholesterol, [1,2,3-$^{13}C_{3}$] myristic acid, [$^{13}C_{5}$] proline, and [$^{13}C_{4}$] disodium -ketoglutarate, obtained from Cil (Andover, MA, USA). In the LC-MS, the internal standard used were [$^{13}C_{9}$] phenylalanine, [$^{13}C_{3}$] caffeine,[D_{4}] cholic acid, [D_{8}] arachidonic acid, and [$^{13}C_{9}$] caffeic acid, obtained from Sigma (St. Louis, MO, USA).
