# Supplementary material for: Why stay in a bad relationship? The effect of local host phenology on a generalist butterfly feeding on a low-ranked host
Source: BMC Evol Biol. 2016 Jun 29;16:144. doi: 10.1186/s12862-016-0709-x (PMC4928354; doi:10.1186/s12862-016-0709-x)
Supplement: Additional file 2: — ANOVA table showing the effect of diet and seasonality on growth rate to emergence. (PDF 85 kb) [file 12862_2016_709_MOESM2_ESM.pdf]

Additional file 2. Type II ANOVA table showing the effect of diet and seasonality on growth rate to emergence (GR). R^2adj=0.55, the residuals are normally distributed.

GR	Sum Sq	df	F	*P*
Diet	0.049	1	77.2	<0.001
seasonality	0.017	1	26.8	<0.001
Residuals	0.074	117		
