# Supplementary material for: Why stay in a bad relationship? The effect of local host phenology on a generalist butterfly feeding on a low-ranked host
Source: BMC Evol Biol. 2016 Jun 29;16:144. doi: 10.1186/s12862-016-0709-x (PMC4928354; doi:10.1186/s12862-016-0709-x)
Supplement: Additional file 3: — ANOVA table showing the effect of diet, family, and survival on frass weight. (PDF 93 kb) [file 12862_2016_709_MOESM3_ESM.pdf]

Additional file 3. Type II ANOVA table showing the effect of diet, family, and survival on the logarithm of frass weight (ln mg). R^2adj=0.91, the residuals are normally distributed.

Ln (Frass weight)	Sum Sq	df	F	P
Diet	4.85	1	36.6	<0.001
Survived to 5th	0.75	1	5.6	0.027
Family	8.48	6	10.6	<0.001
Diet:Family	2.59	6	3.2	0.020
Residuals	2.93	22		
