# Supplementary material for: Why stay in a bad relationship? The effect of local host phenology on a generalist butterfly feeding on a low-ranked host
Source: BMC Evol Biol. 2016 Jun 29;16:144. doi: 10.1186/s12862-016-0709-x (PMC4928354; doi:10.1186/s12862-016-0709-x)
Supplement: Additional file 4: — ANOVA table showing the effect of diet, seasonality and the interaction between diet and seasonality on survival. (PDF 76 kb) [file 12862_2016_709_MOESM4_ESM.pdf]

Additional file 4. Type II ANOVA table showing the effect of diet, seasonality and the interaction between diet and seasonality on survival. The residuals are not normally distributed. This was due to two outlier families. Removing them did not change model selection but it made the residuals normally distributed and removed the overdispersion.

Survival	LR Chisq	df	*P*
Diet	82.9	1	<0.001
seasonality	1.22	1	0.27
Diet:seasonality	8.43	1	<0.01
